# Shortwave-infrared (SWIR) emitting annexin V for high-contrast fluorescence molecular imaging of tumor apoptosis in living mice†

**DOI:** 10.1039/d2ra03315a

**Published:** 2022-07-06

**Authors:** Mahadeva M. M. Swamy, Setsuko Tsuboi, Yuta Murai, Kenji Monde, Takashi Jin

**Affiliations:** Center for Biosystems Dynamics Research, RIKEN Furuedai 6-2-3 Suita Osaka 565-0874 Japan tjin@riken.jp; Graduate School of Life Science, Hokkaido University Kita 21 Nishi 11 Sapporo Hokkaido 001-0021 Japan

## Abstract

Recently, shortwave infrared (SWIR) fluorescence imaging over 1000 nm has attracted much attention for *in vivo* optical imaging because of the higher signal to background ratios in the SWIR region. For the application of SWIR fluorescence imaging to biomedical fields, the development of SWIR fluorescent molecular probes with high biocompatibility is crucial. Although many researchers have designed a variety of SWIR emitting probes based on organic dyes, the synthesis of biocompatible SWIR fluorescent molecular imaging probes is still challenging. In this work we synthesized indocyanine green (ICG) and π-conjugation extended ICG (ICG-C11) labelled annexin V as SWIR fluorescent probes for tumor apoptosis. Annexin V is an endogenous protein with binding ability to phosphatidylserine (PS) which appears on the outer monolayer of apoptotic cell membranes. Although there are many types of visible and NIR fluorescent annexin V, there are no SWIR emitting fluorescent probes that can be used for high contrast fluorescence imaging of apoptosis *in vivo*. Herein, we report the synthesis and application of ICG and ICG-C11 conjugated annexin V for SWIR fluorescence imaging of tumor apoptosis. The presented fluorescent annexin V is the first SWIR emitting probe for *in vivo* optical imaging of tumor apoptosis. We demonstrate that SWIR emitting ICG- and ICG-C11 conjugated annexin V enable high-contrast fluorescence imaging of tumor apoptosis in living mice. We further demonstrate that ICG-C11-annexin V can be used for long-term (*ca.* two weeks) SWIR fluorescence imaging of tumor apoptosis. The SWIR fluorescent annexin V will greatly contribute not only to the study of tumor-apoptosis induced by anti-cancer drugs, but also to the study of apoptosis-related diseases in a living system.

## Abstract 2

The labelling of annexin V with indocyanine green (ICG) and π-conjugation extended ICG (ICG-C11) resulted in SWIR emitting probes that enable high-contrast molecular imaging of tumor apoptosis in living mice.

## Graphical abstract

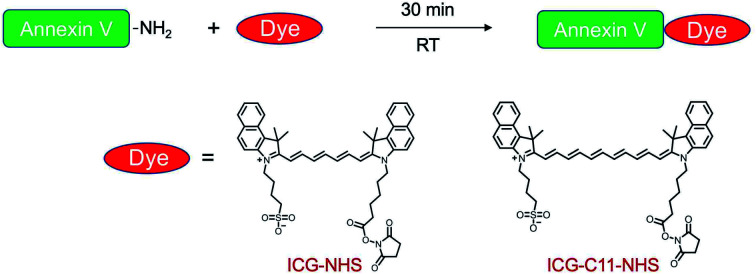

## Introduction

*In vivo* imaging of apoptosis enables monitoring of tumor responses to anti-cancer drugs such as antibody–drug conjugates (ADCs).^1^ For apoptosis imaging, several imaging modalities such as single photon emission computed tomography,^2^ positron emission tomography,^3^ magnetic resonance imaging,^4^ and optical imaging^5^ have been employed in pre-clinical and clinical practice.^6^ Among these imaging modalities, optical imaging has a highest spatial resolution with a high sensitivity sufficient to perform molecular imaging in a living system. To date, near infrared (NIR) fluorescence imaging in the wavelengths from 700 to 900 nm has been used for *in vivo* optical imaging.^7^ Recently, shortwave infrared (SWIR) fluorescence imaging beyond 1000 nm has attracted much attention for *in vivo* optical imaging because of the higher signal to background ratios in the SWIR region.^8,9^ For the application of SWIR fluorescence imaging to biomedical fields, the development of SWIR fluorescent molecular imaging probes with high biocompatibility is crucial. Although many researchers have designed a variety of SWIR emitting fluorescent probes,^8^ the synthesis of biocompatible SWIR fluorescent molecular imaging probes is still challenging.^10,11^

In this work, we have synthesized biocompatible SWIR fluorescent molecular imaging probes based on annexin V for the detection of tumor apoptosis *in vivo*. Annexin V is an endogenous protein with binding ability to phosphatidylserine (PS) which appears on the outer monolayer of apoptotic cell membrane.^12^ This protein has been employed to the construction of fluorescent probes for the detection of apoptosis. For instance, fluorescein conjugated annexin V (FITC-annexin V) is widely used as an optical probe for the detection of apoptosis by fluorescence microscopy and fluorescence-activated cell sorting methods.^13^ Although many types of visible and NIR fluorescence labelled annexin V have been developed,^5,14^ there are no SWIR fluorescent probes that can be used for high-contrast fluorescence imaging of apoptosis *in vivo*. Optical imaging using SWIR fluorescent probes is expected to offer clearer deep tissue images due to the lower light absorption and scattering in the SWIR region.^15^

In this paper, we present indocyanine green^16^ (ICG) and π-conjugation extended ICG conjugated annexin V as SWIR fluorescent probes for tumor apoptosis. Although ICG has an emission maximum of around 830 nm, it still emits in the SWIR region.^17–22^ Several groups have demonstrated the capability of ICG as a SWIR fluorescent probe for *in vivo* imaging of blood vasculatures, lymph systems, and tumors.^15–22^ The disadvantage of ICG for its use in SWIR fluorescence imaging is the limitation of excitation wavelengths of less than 800 nm. To achieve higher contrast fluorescence imaging, longer excitation/emission wavelengths are desirable because of the lower tissue autofluorescence and light scattering. Therefore, we also synthesized a π-conjugation extended ICG (ICG-C11)^11^ conjugated annexin V. ICG-C11 conjugated annexin V has a fluorescence maximum above 1000 nm. Using ICG and ICG-C11 conjugated annexin V, we performed SWIR fluorescence imaging of breast tumor apoptosis induced by treatment of an ADC, trastuzumab emtansine (Kadcyla).^23^ In this study, we demonstrate that ICG and ICG-C11 conjugated annexin V can be used not only for high-contrast SWIR fluorescence imaging of tumor apoptosis, but also for long-term (*ca.* two weeks) imaging of tumor apoptosis in living mice.

## Results and discussion

### Preparation of SWIR emitting annexin V

**Scheme 1:**
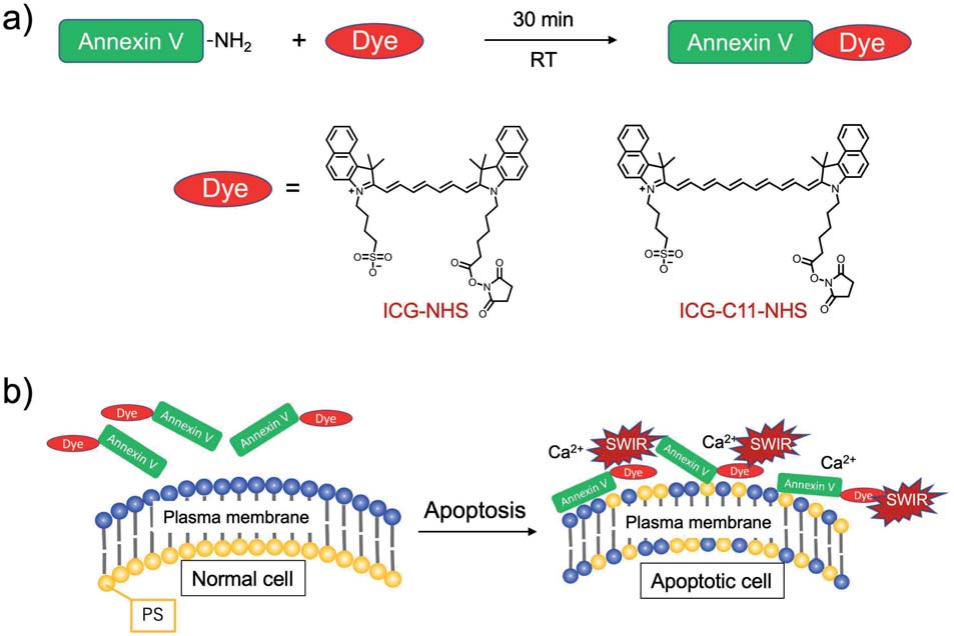
(a) Fluorescence labelling of annexin V with ICG and ICG-C11, where their succinimidyl ester derivatives are used as labelling agents. (b) Binding of SWIR fluorescent-dye labelled annexin V to phosphatidylserine (PS) on the apoptotic cell surface in the presence of Ca^2+^ ions.

To prepare SWIR fluorescent annexin V, we used succinimide activated SWIR fluorescent dyes, ICG-NHS (*N*-hydroxy succinimide ester of ICG) and ICG-C11-NHS^11^ (*N*-hydroxy succinimide ester of ICG-C11) for fluorescence labelling. This type of fluorescent dyes easily binds to primary amino groups of annexin V (Scheme 1a).^24^ ICG-C11 is a π-conjugation extended ICG analogue, where the length of the polymethine chain of ICG-C11 increases by two double bonds compared with that of ICG.^10^

Recently, we reported that ICG-annexin V can act as a NIR fluorescent dye for cancer cell apoptosis *in vitro* and *in vivo*.^22^ ICG and ICG-C11 conjugated annexin V (ICG-annexin V and ICG-C11-annexin V) recognize PS on the plasma membrane of apoptotic cells, and they emit SWIR fluorescence over 1000 nm (Scheme 1b). Using the annexin V probes, we examined the performance of SWIR fluorescence for high-contrast molecular imaging of tumor apoptosis in living mice.

### Optical property of SWIR emitting annexin V

**Fig. 1:**
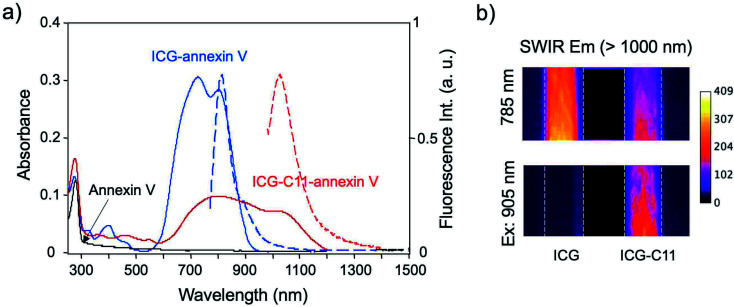
(a) Absorption and fluorescence spectra of annexin V (black), ICG-annexin V (blue) and ICG-C11-annexin V (red). Absorption and fluorescence are depicted as solid lines and broken lines, respectively. (b) SWIR fluorescence images of aqueous solutions of ICG and ICG-C11, which are taken through a long pass filter (>1000 nm) with excitation at 785 nm and 905 nm.

The conjugation of annexin V with ICG and ICG-C11 was confirmed by the measurement of absorption spectra. In the absorption spectra of ICG-annexin V and ICG-C11-annexin V in phosphate buffered saline (PBS), two absorption bands resulting from annexin V protein (*λ*_max_ = 280 nm) and cyanine dye (*λ*_max_ = 727 nm for ICG-annexin V and *λ*_max_ = 800 nm for ICG-C11-annexin V) were observed (Fig. 1a). From the absorption spectra, the number of dye molecules bound to one annexin V protein was calculated to be 0.33 for ICG-annexin V and 0.38 for ICG-C11-annexin V (ESI†).

Fluorescence spectra of ICG-annexin V and ICG-C11-annexin V in PBS (1% bovine serum albumin) showed fluorescence maximum at 813 nm and 1030 nm (Fig. 1a), respectively. In case of ICG-annexin V, it still emitted in the SWIR region over 1000 nm. Fluorescence images of ICG and ICG-C11 in PBS showed that the excitation at 785 nm results in SWIR fluorescence emission both from ICG and ICG-C11 (Fig. 1b), while the excitation at 905 nm results in SWIR fluorescence emission only from ICG-C11 (Fig. 1b).

### Binding activity of SWIR emitting annexin V

To conform the binding activity of ICG-annexin V and ICG-C11-annexin V to apoptotic cells, we performed cellular imaging using a human breast cancer cell line (KPL-4).^25^ Apoptosis of KPL4 cells was induced by Kadcyla. Kadcyla is a conjugate between a humanized monoclonal anti-HER2 (human epidermal growth factor receptor 2)^26^ antibody and maytansinoid (DM1), a highly potent microtubule polymerization inhibitor.^25^ Kadcyla is used as an anti-cancer drug against HER2 positive cancer.^27^

**Fig. 2:**
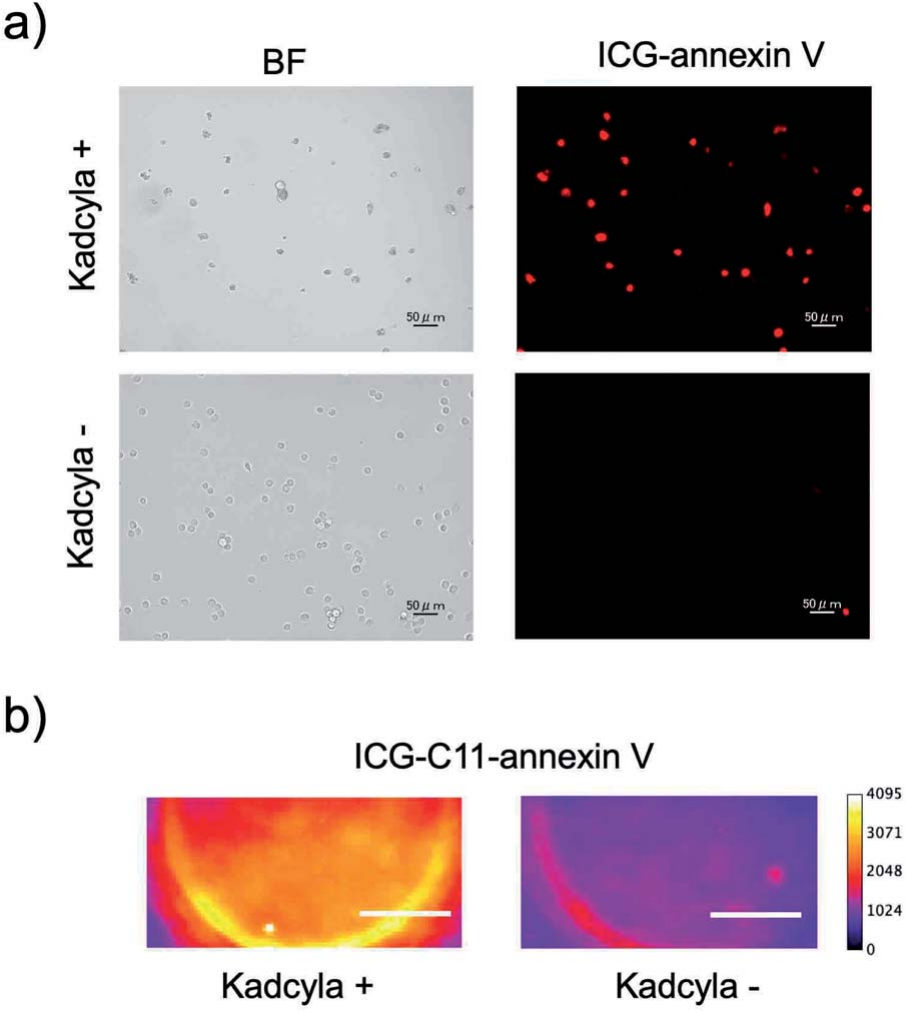
(a) NIR fluorescence imaging of KPL-4 cells stained with ICG-annexin V. The KPL-4 cells were incubated three days with Kadcyla (upper image) and without Kadcyla (lower image). NIR fluorescence was observed through a band path filter (832 ± 19 nm) with excitation at 769 ± 20 nm. Scale bar: 50 μm. (b) SWIR fluorescence imaging of the KPL-4 cell pellets incubated with Kadcyla (left image) for three days and without Kadcyla (right image). The SWIR fluorescence images were taken through a long pass filter (>1000 nm) with excitation at 905 nm. Scale bar: 1 mm.

After the treatment of KPL-4 cells with Kadcyla for three days, fluorescence imaging was performed using ICG-annexin V and ICG-C11-annexin V (Fig. 2). Cellular imaging showed that KPL-4 cells incubated with ICG-annexin V emit significant NIR fluorescence from the cells (upper image in Fig. 2a), indicating that the apoptosis of KPL-4 cells was induced by Kadcyla (Fig. S1†).‡‡Cellular imaging using FITC-annexin V also confirmed the induction of apoptosis of KPL-4 cells by Kadcyla. In contrast, the control cells with incubation of no Kadcyla did not show significant fluorescence emission from KPL-4 cells (lower image in Fig. 2a). To examine the binding activity of ICG-C11-annexin V, we also performed SWIR fluorescence imaging for the cell pellets of KPL-4. SWIR fluorescence imaging of the cell pellets showed a similar result as observed in the cellular imaging using ICG-annexin V. SWIR fluorescence emission was observed for the KPL-4 cell pellets treated with Kadcyla, while no significant SWIR fluorescence emission was observed for the control cells without Kadcyla (Fig. 2b). Cell viability assay showed that ICG-annexin V and ICG-C11-annexin V have very low cytotoxicity in the concentrations up to 1 μM (Fig. S2†).

### NIR and SWIR fluorescence imaging of ICG-annexin V

For *in vivo* imaging of tumor apoptosis, we used HER2-positive breast-tumor bearing mice. Breast-tumor bearing mice were prepared by implanting of KPL-4 cells to nude mice. Tumor apoptosis in the nude mice was induced by Kadcyla. Using SWIR emitting annexin V probes, we achieved *in vivo* imaging of the apoptosis of HER2-positive breast tumors.

**Fig. 3:**
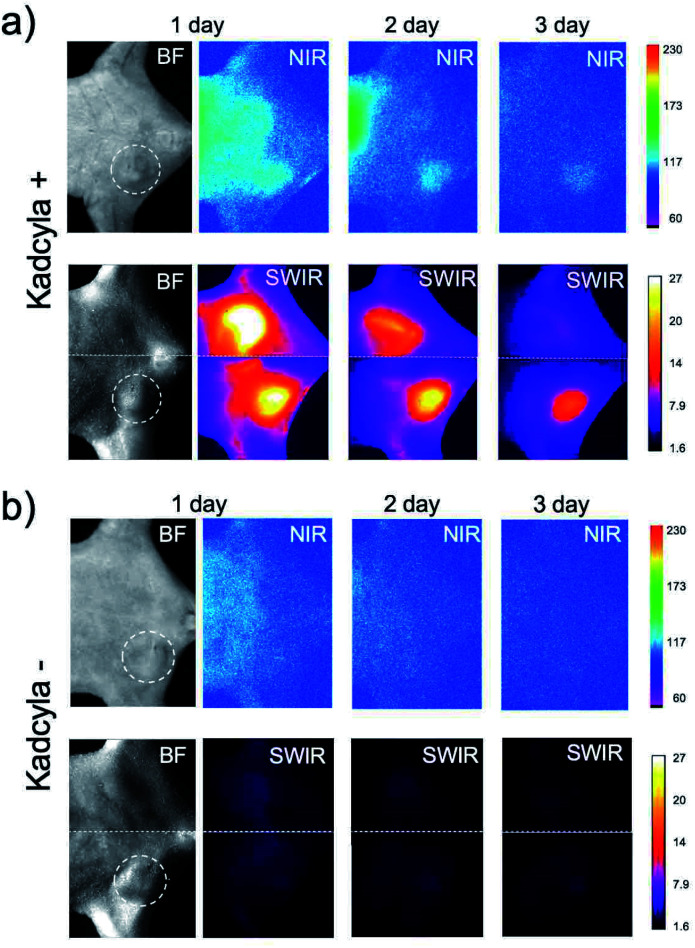
Bright filed (BF) and NIR/SWIR fluorescence imaging of breast-tumor bearing mice injected with Kadcyla (a) and without Kadcyla (b). Kadcyla was intravenously injected to the mice three days before the injection of ICG-annexin V. White dotted lines show the position of a breast tumor in the mouse. Fluorescence images were taken one, two, and three days after the intravenous injection of 200 μL of ICG-annexin V (1 mg mL^−1^ PBS solution). NIR fluorescence was detected using a band path filter (830 ± 15 nm) with excitation at 760 nm. SWIR fluorescence was detected using a long pass filter (>1000 nm) with excitation at 785 nm.

Intravenous injection of ICG-annexin V resulted in NIR and SWIR fluorescence emissions from the tumors (Fig. 3a), showing the accumulation of ICG-annexin V to the tumor, where apoptosis was induced by Kadcyla (Fig. S3†).§§The uptake of Kadcyla to KPL-4 cells was confirmed by *in vitro* and *in vivo* imaging using Alexa 680-labelled Kadcyla. Three days after the injection of ICG-annexin V, the accumulation of ICG-annexin V to the tumor was clearly observed (right images in Fig. 3a and S4†). In contrast, significant NIR and SWIR fluorescence of ICG-annexin V was not observed from the tumor of a control mouse without Kadcyla (Fig. 3b). In case of a Kadcyla treated mouse, SWIR fluorescence images were much clearer than NIR fluorescence images (Fig. 3a). The signal to background ratio of 3 day fluorescence image of a tumor was estimated to be 1.1 for NIR and 7.2 for SWIR, showing the higher contrast of SWIR fluorescence imaging compared with NIR fluorescence imaging. This is explained by the lower tissue autofluorescence in the SWIR region.^9^

### SWIR fluorescence imaging of ICG-C11-annexin V

**Fig. 4:**
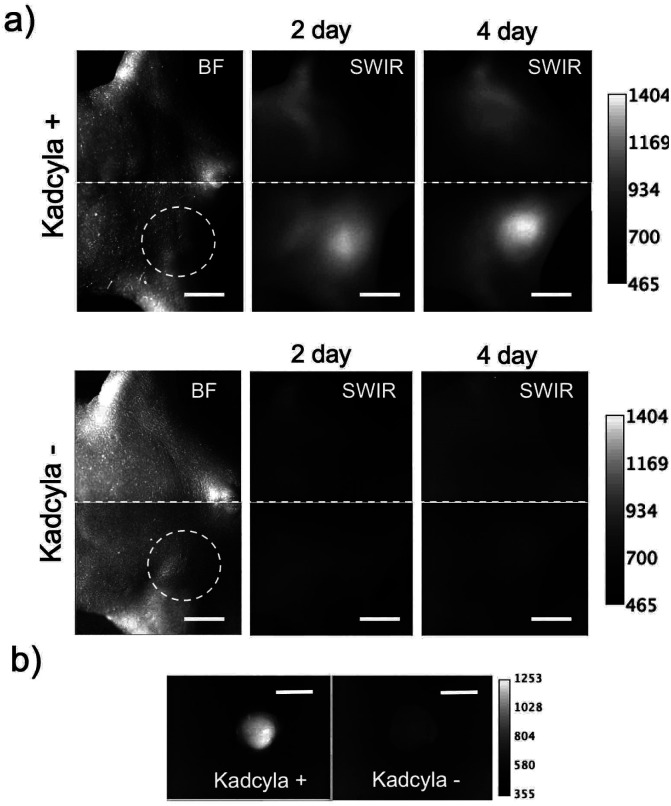
(a) Bright field (BF) and SWIR fluorescence imaging of breast tumor bearing mice injected with Kadcyla (upper image) and without Kadcyla (lower image). Dotted lines show the position of a breast tumor in the mouse. Kadcyla was intravenously injected to the mice three days before the injection of ICG-C11-annexin V. Fluorescence images were taken two and four days after the intravenous injection of 200 μL of ICG-C11-annexin V (1 mg mL^−1^ PBS solution). Scale bar: 5 mm. (b) *Ex vivo* SWIR fluorescence imaging of breast tumors which were isolated from the above mice.

To examine the performance of SWIR fluorescence for *in vivo* imaging, we further performed fluorescence imaging of tumor apoptosis using ICG-C11-annexin V. As the case of ICG-annexin V, ICG-C11-annexin V accumulated to apoptotic tumors in living mice. We observed intense SWIR fluorescence of ICG-C11-annexin V from a breast tumor, where the apoptosis was induced by Kadcyla (upper images in Fig. 4a). A control imaging experiment showed no significant SWIR fluorescence emission from a breast-tumor mouse injected with no Kadcyla (lower images in Fig. 4a). *Ex vivo* fluorescence imaging of a breast tumor and organs clearly showed the accumulation of ICG-C11-annexin V to the apoptotic tumor (Fig. 4b). The fluorescence intensity of SWIR fluorescence emission from the tumor (Kadcyla+) was 1.8 times higher than that of the control tumor (Kadcyla−).

### Long-term SWIR fluorescence imaging of tumor apoptosis

**Fig. 5:**
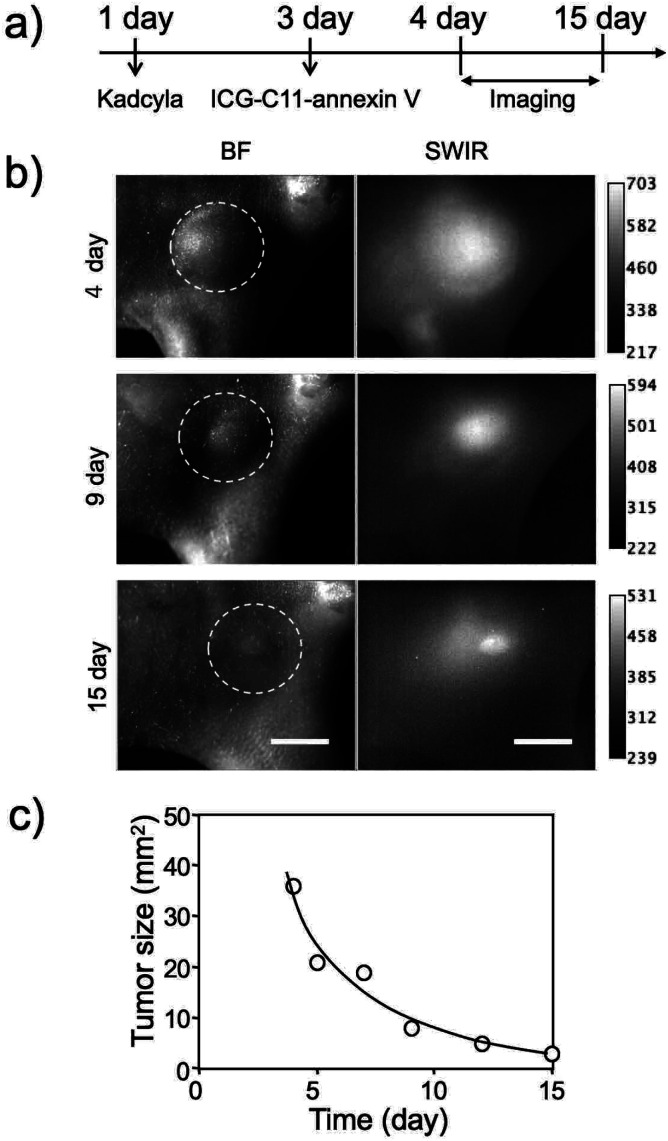
(a) Time course of experimental procedure. ICG-C11-annexin V was injected to the mouse three days after the injection of Kadcyla. Fluorescence images were taken from 4 to 15 days after the intravenous injection of 200 μL of Kadcyla (1 mg mL^−1^ PBS solution). (b) Bright field (BF) and SWIR fluorescence imaging of an apoptosis-induced breast tumor by Kadcyla. Dotted lines show the position of a breast tumor in the mouse. SWIR fluorescence: >1000 nm, Ex: 905 nm. Scale bar: 5 mm. (c) Time course of the change in the tumor size in the above mouse.

Finally, we examined whether ICG-C11-annexin V can be employed to long-term fluorescence imaging of tumor apoptosis. SWIR fluorescence imaging was performed for a breast tumor-bearing mouse injected with Kadcyla (Fig. 5a). SWIR fluorescence images were taken at 4 to 15 days after the injection of Kadcyla. Nine days after the injection of Kadcyla, the significant shrinking of the tumor (from 9 to 3 mm in diameter) was observed (Fig. 5b). The size of the breast tumor was decreased by three times 15 days after the injection of Kadcyla, showing the significant effect of the anti-cancer drug, Kadcyla, on the shrinking of the breast tumor (Fig. 5a and b and S5†).

## Experimental

### Materials and methods

The solvents and chemicals used for the synthesis of ICG-NHS (ESI), ICG-C11,^10^ and ICG-C11-NHS^10^ were purchased from Sigma-Aldrich, TCI (Tokyo, Japan) and FUJIFILM Wako Pure Chemical Corporation, Japan. The reactions were monitored by thin-layer chromatography (TLC) on silica gel plates (0.2 mm, Merck 60 F254 which were visualized with UV light. SiO_2_ gel column chromatography was carried out using silica gel (Wakogel® N60, spherical, 38–100 μm). Indocyanine green (ICG) was purchased from Sigma-Aldrich. ICG-C11 and ICG-C11-NHS were synthesized according to the previously reported methods.^10^ Fluorescein isothiocyanate (FITC) was purchased from Molecular Probes. *N*-Succinimidyl ester derivative of Alexa 680 (Alexa Fluor™ 680 NHS ester), Alexa 680-NHS was purchased from Thermo Fisher Scientific. Kadcyla was purchased from Chugai Pharmaceutical Co., Ltd (Tokyo, Japan).

Breast tumor cells were kindly provided by Dr Kurebayashi (Kawasaki Medical School). Nude mice (five-week-old female BALB/c nu/nu mice) were purchased from Nihon SLC Inc. Mice maintenance and animal experiments were performed in accordance with the Guidelines for Care and Use of Laboratory Animals of RIKEN and approved by the Animal Ethics Committee of RIKEN.

Absorption spectra were recorded with a spectrophotometer (V-670, Jasco). Fluorescence spectra were recorded with a spectrofluorometer (Nanolog, Horiba, Japan). Cellular imaging was performed with a fluorescence microscope (BZ-X700, Keyence). Flow cytometric analysis was performed using MACSQuant analyzer (Miltenyi Biotec Inc.) NIR and SWIR fluorescence imaging was performed with a *in vivo* fluorescence imaging system (Bruker MS-FX PRO) and a lab-made SWIR fluorescence imaging system, respectively.^9^

ICG-NHS structure was characterized by ^1^H NMR (500 MHz) measured on a Varian Inova instrument in DMSO-d_6_ at 25 °C. Chemical shifts (*δ*) are described in ppm relative DMSO-d_6_ (*δ* 2.50) and tetramethylsilane (*δ*: 0) and coupling constant values (*J*) are described in Hertz (Hz). The following abbreviations are used for signal multiplicities: s = singlet; d = doublet; t = triplet; m = multiplet, and dd = doublet of doublet. High resolution mass spectra (HRMS) were obtained using Q-Exactive Orbitrap instrument (Thermo Fisher Scientific, Massachusetts, USA).

### Synthesis of annexin V

Annexin V sequence was amplified by PCR from pET12a-PAPI, which was a gift from Jonathan Tait (Addgene plasmid #19961, B. L. Wood, D. F. Gibson, and J. F. Tait, Blood 1996, 88, 1873–1880). The PCR fragment was fused with pRSET plasmid (ThermoFisher) by using an InFusion HD cloning kit (Clontech).

The pRSET–Annexin V plasmid was transformed into *E. coli* KRX competent cells (Promega). Transformed *E. coli* was grown as a preculture 2 mL of LB medium containing ampicillin (100 mg mL^−1^) at 37 °C overnight. For large-scale culture, the overnight culture (2 mL) was grown in 200 mL of LB medium containing ampicillin (100 mg mL^−1^) at 37 °C, until they approached to 0.6 of OD 600 (absorbance). To induce production of the targeted protein, isopropyl β-d-thiogalactopyranoside (0.2 mM) and l-rhamnose (0.1%) were added to the LB medium, and then incubated with shaking gently for 16 hours at 18 °C.

The cells were harvested by centrifugation at 5000*g* for 10 minutes. The pellet was resuspended in 5 mL of binding buffer (50 mM Tris–HCl, 500 mM NaCl, 20 mM imidazole, pH 8.0). Before cell lysis, complete EDTA-free protease inhibitor cocktail tablets (1×, Roche) were added as a protease inhibitor. The suspension was sonicated on ice. Bursts of 10 seconds with intermediate intensity are repeated 7–10 times with a 10 second cooling period between each burst. The lysate was clarified by centrifugation at 20 000*g* for 30 minutes to eliminate cell debris. The supernatant was then purified by Ni Sepharose 6 Fast Flow (GE Healthcare). 0.5 mL of Sepharose media equilibrated with binding buffer was added to 5 mL of lysed sample and incubated with gentle agitation at 4 °C for 60 minutes. After the solution was transferred to an empty column, it was washed with binding buffer five column volumes. Lastly, the recombinant proteins were drained from the column by the addition of elution buffer (50 mM Tris–HCl, 500 mM NaCl, 500 mM imidazole, pH 8.0). The eluted fractions were further purified by a size-exclusion column (PD-10, GE Healthcare) to exchange buffer.

### Fluorescence labelling of annexin V with ICG-NHS and ICG-C11-NHS

ICG-NHS (1 mg), ICG-C11-NHS (1 mg), or FITC (1 mg) was resolved to 1 mL of anhydrous dimethyl sulfoxide. To an aqueous solution (0.01 M Na_2_CO_3_) of annexin V (1 mg mL^−1^), 30 μL of a dimethyl sulfoxide solution of ICG-NHS, ICG-C11-NHS or FITC was dropwisely added. The coupling reaction with ICG-NHS and ICG-C11-NHS was performed for 30 min at room temperature. The coupling reaction with FITC was performed for overnight at 4 °C. The dye-annexin V conjugates were purified by using a size-exclusion column (PD-10, GE Healthcare) to remove unreacted dyes. The degree of labeling of the purified dye-annexin V was determined by measuring the absorbance of the conjugates in PBS buffer. The molar extinction coefficient of annexin V (24 870 m^−1^ cm^−1^ at 280 nm) was calculated using ProtParam.^28^ The molar extinction coefficients of ICG (780 nm) and ICG-C11 (800 nm) were 170 000 and 54 800, respectively.^10^

### Fluorescence labeling of Kadcyla with alexa 680 and ICG-C11

Alexa 680-NHS (1 mg) was resolved to 1 mL of anhydrous dimethyl sulfoxide. To an aqueous solution (0.01 M Na_2_CO_3_) of Kadcyla (1 mg mL^−1^), 30 μL of a dimethyl sulfoxide solution of Alexa 680-NHS was dropwisely added. The coupling reaction was performed for 30 min at room temperature. The alexa 680 conjugated Kadcyla (alexa 680-Kadcyla) was purified by using a size-exclusion column (PD-10, GE Healthcare) to remove unreacted dyes.

### Cellular imaging and flow cytometric analysis

KPL-4 cells were plated to 35 mm cell culture dishes (3 × 10^5^ cells per dish) and incubated in Dulbecco's modified Eagle Medium supplemented with 10% fetal bovine serum, 100 mg mL^−1^ penicillin, and 10 mg mL^−1^ streptomycin in 5% CO^2^ at 37 °C for overnight. Then, the cells were incubated with Kadcyla (10 nM or none) for 72 hours. All the cells floating in the medium and the cells that had detached during the PBS washing were collected, and the cells attached to the dish were carefully detached by trypsinization and added to the cell suspension. The cells were washed once with PBS and then resuspended in 1 mL of Annexin V binding buffer (Nacalai Tesque). The cell suspension was divided into 100 μL aliquots, added FITC–Annexin V (5 μL; FITC–Annexin V Apoptosis Detection Kit, Nacalai Tesque) or ICG–Annexin V (final concentration, 1.3 μM) and incubated at room temperature for 15 minutes in the dark. Then, 400 μL of binding buffer was added to the stained cell suspension, which was passed through a 35 μm cell strainer before use in the next experiment. Quantification by flow cytometry was performed using the MACSQuant Analyzer (Miltenyi Biotec Inc.). Fluorescence of FITC was collected through a FL2 filter (ex: 488 nm, em: 525 ± 25 nm). Fluorescence images were acquired with a fluorescence microscope (BZ-X700, Keyence Corp., Japan). The filter set for FITC was ex: 470 ± 20 nm, em: 525 ± 25 nm. The filter set for ICG was ex: 769 ± 20 nm, em: 832 ± 19 nm.

For SWIR fluorescence imaging, KPL-4 cells were incubated with Kadcyla (10 nM or none) for 72 hours and then collected as above. Next, the cells were added with ICG-C11-Annexin V (final concentration, 1 μM) and incubated at room temperature for 15 minutes in the dark. After three PBS washes, the cells were resuspended in 20 μL PBS and placed in 3 mm diameter wells for observation. SWIR fluorescence imaging of the cell pellets were acquired with a lab-made fluorescence imaging system. The filter set for ICG-C11 was ex: 905 nm, em: >1000 nm LP.

To confirm the interaction of cells and Kadcyla, KPL-4 and HeLa cells were incubated with 1 μM Alexa 680-Kadcyla at 37 °C for 10 minutes. After three PBS washes, fluorescent images were observed under a fluorescence microscope. The filter set for Alexa 680 was ex: 620 ± 30 nm, em: 700 ± 38 nm. The cells were then detached by trypsinization and analyzed by flow cytometry. Fluorescence of Alexa 680 was collected through a FL6 filter (ex: 635 nm, em: 692 ± 38 nm).

### Cytotoxicity test

KPL-4 cells were incubated with ICG-Annexin V and ICG-C11-Annexin V (0–1 μM) for 6, 24, and 48 hours. The MTT assay was performed according to the procedure provided by the MTT Cell Count Kit (Nacalai Tesque). MTT reagent was added to each well, and the cells were incubated at 37 °C for 2 hours. Next, the STOP solution was added to terminate the reaction, and the absorbance of the solubilized MTT formazan product at 570 nm was measured with a microplate spectrophotometer (Multiskan GO; Thermo Fisher Scientific). Absorbance at 650 nm was subtracted as the background.

### Preparation of tumor-bearing mice

A suspension of KPL-4 cells (10^7^ cells per mouse) was transplanted to the ventral side of 5 weeks old female BALB/c nu/nu mice. After several weeks, we selected a mouse bearing a breast tumor which size is less than 10 mm in diameter for imaging.

### *In vivo* NIR and SWIR fluorescence imaging

For NIR and SWIR fluorescence imaging of tumor-apoptosis, an aqueous solution (200 μL) of Kadcyla (1 mg mL mL^−1^) was intravenously injected to a breast-tumor bearing mouse *via* its tail vein. Three days later, an aqueous solution (200 μL) of ICG-annexin V or ICG-C11-annexin V (1 mg mL^−1^) was intravenously injected to a breast-tumor bearing mouse *via* its tail vein. Fluorescence imaging measurements were started one day after the injection of the annexin V probes. NIR fluorescence was detected at 830 nm with excitation at 760 nm, where the exposure time was 30 s. SWIR fluorescence was detected using a band-path (1000 nm) and a long path filter (>1000 nm), where the exposure time was 1–5 s.

For two-color SWIR fluorescence imaging, a band-path (1000 nm) with excitation at 785 nm and a long path filter (>1050 nm) with excitation at 905 nm were used. For SWIR fluorescence imaging, 785 and 905 nm emitting solid lasers (laser power of 20 mW cm^−2^) were used. Exposure time was 30 s.

For NIR fluorescence imaging of a tumor-bearing mouse injected with alexa 680-Kadcyla, an aqueous solution (200 μL) of alexa 680-Kadcyla (1 mg mL^−1^) was intravenously injected to the mouse *via* its tail vein. NIR fluorescence was detected at 750 nm with excitation of 650 nm.

## Conclusion

In this paper, we have presented the synthesis and applications of ICG and ICG-C11 conjugated annexin V for SWIR fluorescence imaging of tumor apoptosis. The presented fluorescent annexin V is the first probe that can be used for *in vivo* optical imaging of tumor apoptosis in the SWIR region. We demonstrate that SWIR fluorescent ICG-annexin V and ICG-C11-annexin V enable high-contrast fluorescence imaging of tumor apoptosis in living mice. We further demonstrate that ICG-C11-annexin V can be used for long-term (*ca.* two weeks) SWIR fluorescence imaging of tumor apoptosis. We believe that the SWIR fluorescent annexin V proves will greatly contribute not only to the study of tumor-apoptosis induced by anti-cancer drugs, but also to the study of apoptosis-related diseases in a living system.

## Conflicts of interest

There are no conflicts to declare.

## Supplementary Material

RA-012-D2RA03315A-s001
