# Supplementary material for: Shortwave-infrared (SWIR) emitting annexin V for high-contrast fluorescence molecular imaging of tumor apoptosis in living mice
Source: RSC Adv. 2022 Jul 6;12(30):19632–9. doi: 10.1039/d2ra03315a (PMC9257772; doi:10.1039/d2ra03315a)
Supplement: RA-012-D2RA03315A-s001 [file RA-012-D2RA03315A-s001.pdf]

Electronic Supplementary Material (ESI) for RSC Advances.
This journal is © The Royal Society of Chemistry 2022

Electronic supplementary information

Shortwave-infrared (SWIR) emitting annexin V for high-contrast fluorescence molecular imaging of tumor apoptosis in living mice

Mahadeva M. M. Swamy,[a,b] Setsuko Tsuboi,[a] Yuta Murai,[a,b] Kenji Monde,[a,b] and Takashi Jin[a*]

[a] *Center for Biosystems Dynamics Research, RIKEN, Furuedai 6-2-3, Suita, Osaka 565-0874, Japan.*
[b] *Graduate School of Life Science, Hokkaido University, Kita 21 Nishi 11, Sapporo, Hokkaido 001-0021, Japan.*

Table of Contents

1. Synthesis of ICG-NHS
2. **Fig. S1** a) Cellular imaging of FITC-annexin V stained KPL-4 cells incubated with and without Kadcyla.
3. **Fig. S2** Viability of KPL-4 cells in the presence of ICG-annexin V and ICG-C11-annexin V. KPL-4 cells were incubated with ICG-Annexin V and ICG-C11-Annexin V
4. **Fig. S3** a) Cellular imaging of KPL-4 cells (HER2+) and HeLa cells (HER2-) incubated with alexa 680-Kadcyla.
5. **Fig. S4** a) Ex vivo SWIR fluorescence imaging (ICG-C11-anenxin V) of breast tumors of the mice injected with and without Kadcyla. b) Ex vivo SWIR fluorescence imaging of a breast tumor and organs for the mouse treated with Kadcyla.
6. **Fig. S5** a) Experimental procedure for the administration of Kadcyla/ICG-C11-annexin V and imaging. b) Time course of SWIR fluorescence image of a breast tumor in a living mouse.

Synthesis of ICG-NHS

ICG-NHS was synthesized in three steps as shown in a following scheme.

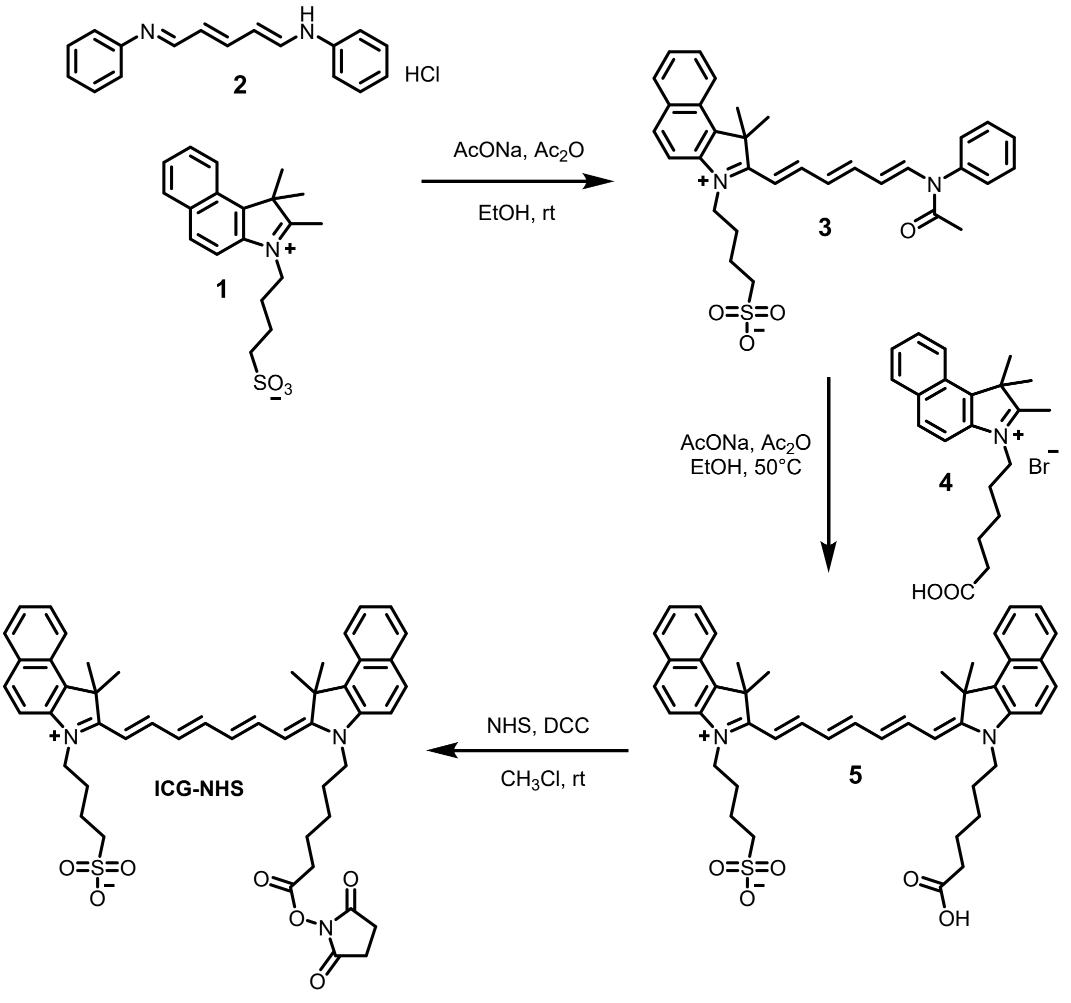

4-(1,1-dimethyl-2-((1*E*,3*E*,5*E*)-6-(*N*-phenylacetamido)hexa-1,3,5-trien-1-yl)-1*H*-benzo[*e*]indol-3-ium-3-yl)butane-1-sulfonate (3) [1]

To a stirred solution of **1** (1 g, 2.89 mmol) and NaOAc (0.71 g, 8.67 mmol) in EtOH (30 mL) at room temperature (rt), acetic anhydride (1.77 g, 17.34 mmol) was added dropwise under inert atmosphere. The reaction mixture was stirred for 10 minutes before adding glutaconaldehydedianil hydrochloride **2** (1.23 g, 4.34 mmol). The mixture was stirred at rt for about 30 minutes. Solvent was removed *in vacuo* and the residue was purified by column chromatography using $CHCl_3$/MeOH (9:1) to obtain compound **3** as dark blue solid (0.86 g, 55%).

4-(2-((1*E*,3*E*,5*E*,7*E*)-7-(3-(5-carboxypentyl)-1,1-dimethyl-1,3-dihydro-2*H*-benzo[*e*]indol-2-ylidene)hepta-1,3,5-trien-1-yl)-1,1-dimethyl-1*H*-benzo[*e*]indol-3-ium-3-yl)butane-1-sulfonate (5)

To a stirred solution of **4** (372 mg, 0.92 mmol) and NaOAc (125 mg, 1.53 mmol) in EtOH (15 mL) at rt, acetic anhydride (103 mg, 1.01 mmol) was added dropwise under inert atmosphere. Compound **3** (500 mg, 0.92 mmol) dissolved in EtOH (10 mL) was added to the reaction mixture. The mixture was stirred at 50 °C for 1.5 to 2 hours. Solvent was removed *in vacuo* and the residue was purified by column chromatography using $CHCl_3$/MeOH (8:2) to obtain **5** as dark green solid (445 mg, 66%).

ICG-NHS

To a stirred solution of compound **5** (200 mg, 0.274 mmol) and NHS (63 mg, 0.548 mmol) in $CHCl_3$ (10 mL) at 0 °C under inert atmosphere, DCC (113 mg, 0.548 mmol) in $CHCl_3$ (1mL) was added dropwise. The reaction mixture was stirred for about 4 hours at rt. Solvent was removed *in vacuo* and the residue was purified by column chromatography using $CHCl_3$/MeOH (9:1) to obtain **ICG-NHS** as dark brown solid (125 mg, 55%).

HRMS (m/z): $[M+H]^+$ calculated for $C_{49}H_{53}N_3O_7S$: 828.3670; found 828.3660

^{1}H NMR (DMSO-d_6 , 500 MHz): δ = 8.24 (2H, t, J = 7.8 Hz), 7.94-8.07 (6H, m), 7.78 (2H, d, J = 9.3 Hz), 7.62-7.69 (3H, m), 7.50 (2H, dd, J = 15.6, 8.3 Hz), 6.52-6.63 (3H, m), 6.39 (1H, d, J = 13.7), 4.22 (2H, t, J = 7.3 Hz), 4.17 (2H, t, J = 6.8 Hz), 3.55 (1H, d, J = 12.6 Hz), 2.80 (3H, s), 2.69 (2H, t, J = 7.3 Hz), 2.59 (1H, s), 2.52-2.54 (1H, m), 1.91 (2H, s), 1.68-1.86 (8H, s), 1.52 (2H, quintet, J = 7.3 Hz).

References

1) T. Doi, K. Oikawa, J. Suzuki, M. Yoshida, N. Iki, Synlett., 2012, 23, 306-310.

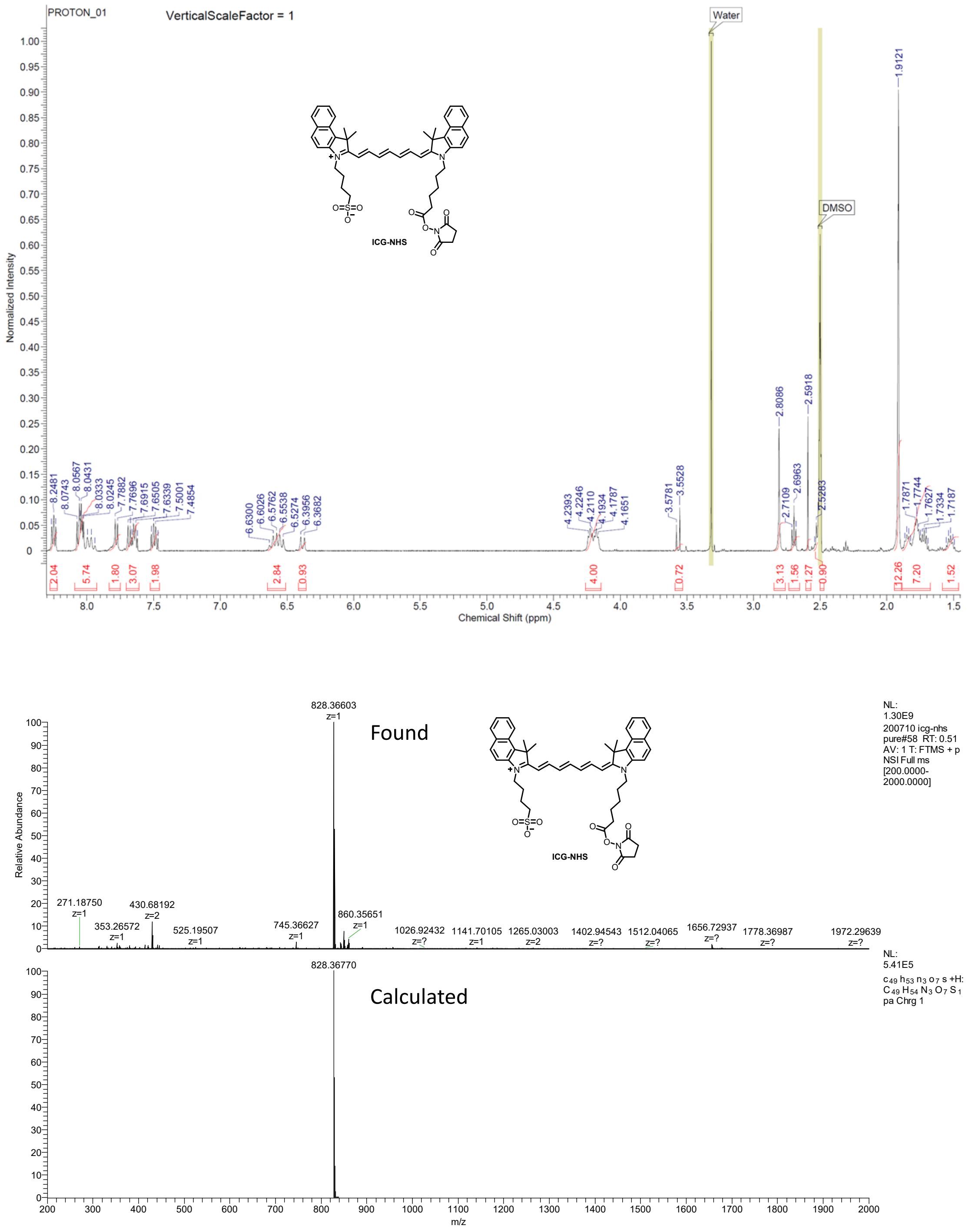

^{1}H-NMR spectra (500 MHz) and HRMS spectra of ICG-NHS.

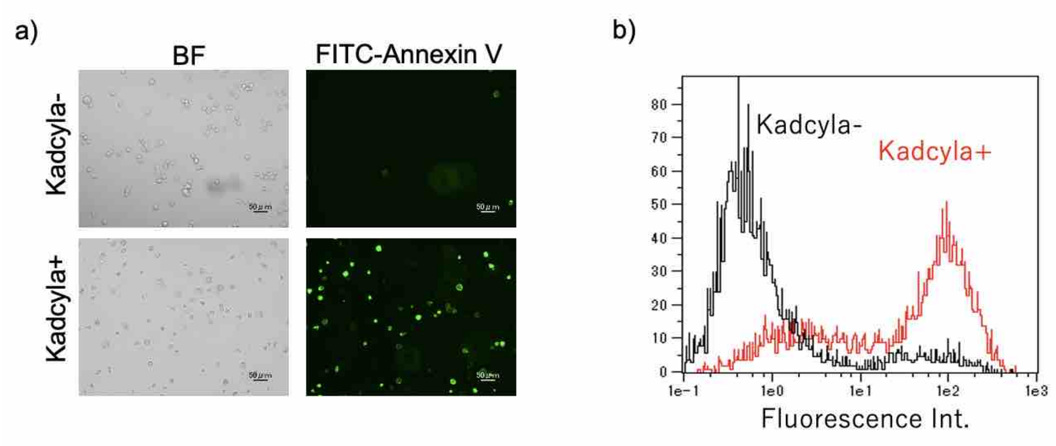

Fig. S1 a) Cellular imaging of FITC-annexin V stained KPL-4 cells incubated with and without Kadcyla. Ex: 470 ± 20 nm, Em: 525 ± 25 nm. Scale bar: 50 μm. b) Flow cytometric analysis of FITC-annexin V stained KPL-4 cells incubated with and without Kadcyla.

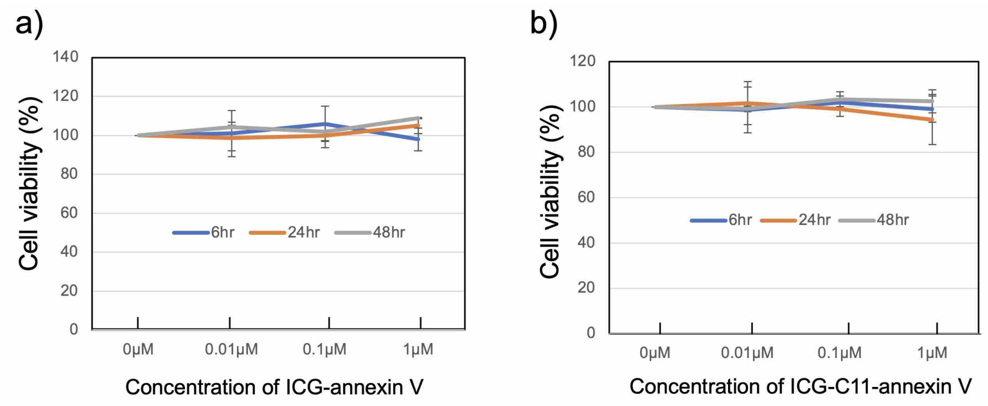

Fig. S2 Viability of KPL-4 cells in the presence of ICG-annexin V and ICG-C11-annexin V. KPL-4 cells were incubated with ICG-Annexin V and ICG-C11-Annexin V (0–1 μM) for 6, 24, and 48 hours.

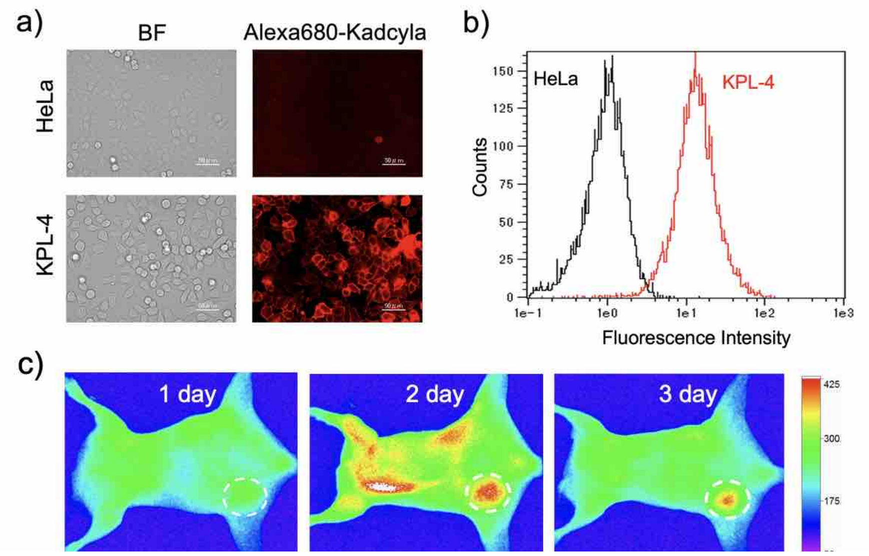

Fig. S3 a) Cellular imaging of KPL-4 cells (HER2+) and HeLa cells (HER2-) incubated with alexa 680-Kadcyla. Scale bar: 50 μm. b) Flow cytometric analysis of KPL-4 cells (HER2+) and HeLa cells (HER2-) incubated with alexa 680-Kadcyla. c) NIR fluorescence imaging of a breast-tumor bearing mouse intravenously injected with alexa 680-Kadcyla. White dotted lines show the position of a breast tumor in the mouse. Images were taken 1, 2, and 3 days after the injection of alexa 680-Kadcyla. Ex: 650 nm, Em: 750 nm.

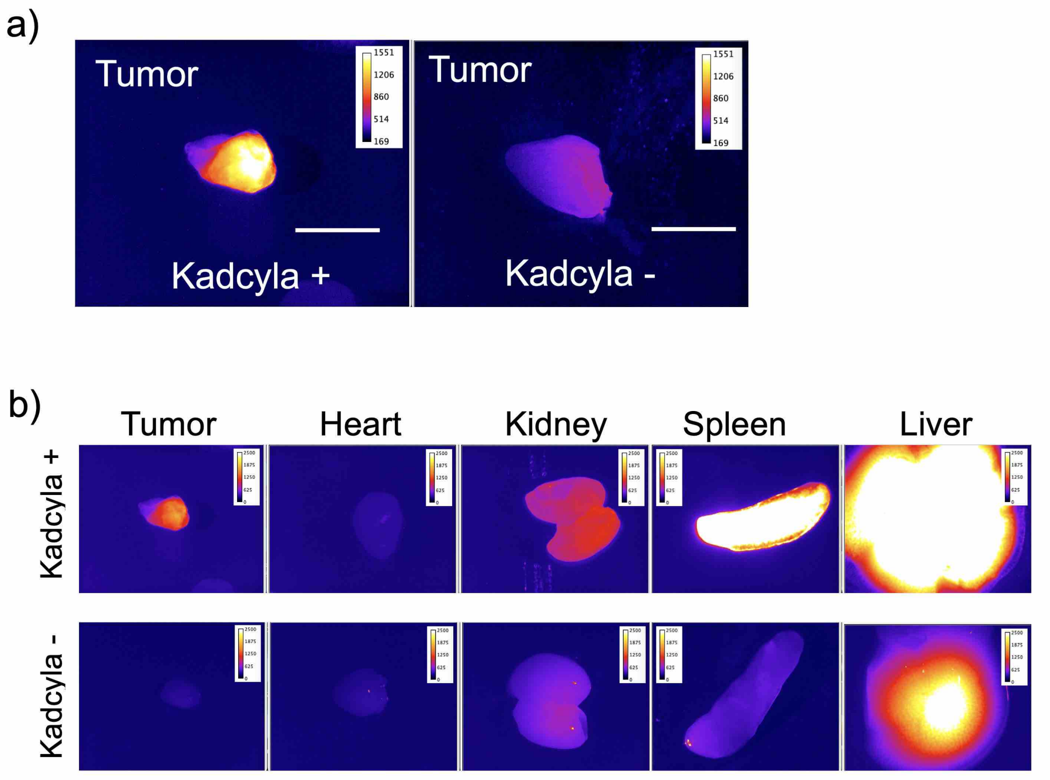

Fig. S4 a) Ex vivo SWIR fluorescence imaging (ICG-annexinV) of breast tumors of the mice injected with and without Kadcyla. Kadcyla was intravenously injected to the mice 3 days before the injection of ICG-annexin V. Fluorescence images of tumors were taken 3 days after the intravenous injection of 200 μL of ICG-annexin V (1mg/mL PBS solution). Scale bar: 5 mm. b) Ex vivo SWIR fluorescence imaging (ICG-annexinV) of a breast tumor and organs for the mouse injected with and without Kadcyla . Ex: 785 nm, Em: >1000 nm.

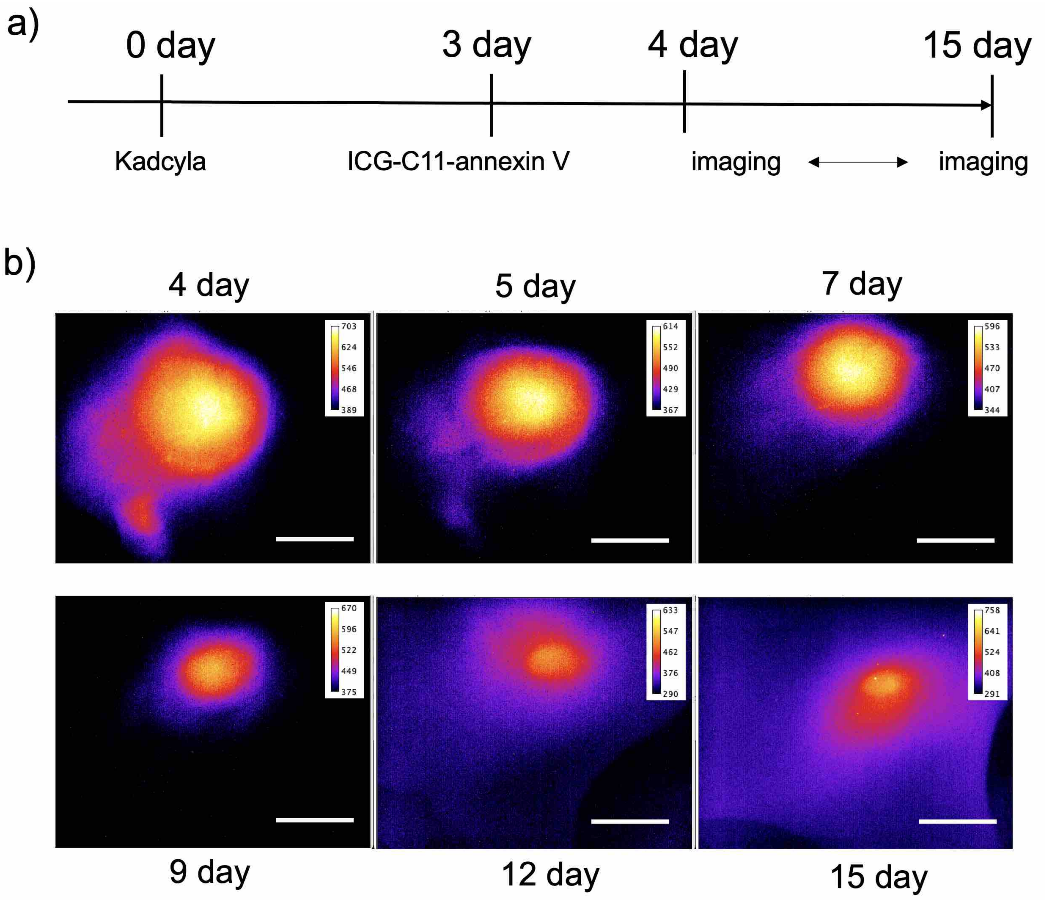

Fig. S5 a) Experimental procedure for the administration of Kadcyla/ICG-C11-annexin V and imaging. Kadcyla was intravenously injected to a HER-2 positive breast tumor bearing mouse. Three days later, ICG-C11-annexin V was injected to the mouse. b) Time course of SWIR fluorescence image of a breast tumor in a living mouse. SWIR fluorescence images were taken through a long pass filter (>1000 nm) with excitation at 905 nm. Laser power: 20 mW/cm^2. Scale bar: 5 mm.
